# 2-Amino-5-chloro­pyridinium 2-carb­oxy­acetate

**DOI:** 10.1107/S1600536810019677

**Published:** 2010-05-29

**Authors:** Madhukar Hemamalini, Hoong-Kun Fun

**Affiliations:** a X-ray Crystallography Unit, School of Physics, Universiti Sains Malaysia, 11800 USM, Penang, Malaysia

## Abstract

The title salt, C_5_H_6_ClN_2_^+^·C_3_H_3_O_4_^−^, contains two cations and two anions in the asymmetric unit. Both 2-amino-5-chloro­pyridinium ions are protonated at their pyridine N atoms and both hydrogen malonate ions feature an intra­molecular O—H⋯O hydrogen bond, which generates an *S*(6) ring motif and results in a folded conformation. In the crystal structure, the cations and anions are linked *via* N—H⋯O, O—H⋯O and C—H⋯O hydrogen bonds, forming chains propagating in [010], which are cross-linked by further C—H⋯O inter­actions.

## Related literature

For background to the chemistry of substituted pyridines, see: Amr *et al.* (2006 ▶); Bart *et al.* (2001 ▶); Shinkai *et al.* (2000 ▶); Klimesôva *et al.* (1999 ▶). For related structures, see: Pourayoubi *et al.* (2007 ▶); Janczak & Perpétuo (2009 ▶); Akriche & Rzaigui (2005 ▶). For details of hydrogen bonding, see: Jeffrey & Saenger (1991 ▶); Jeffrey (1997 ▶); Scheiner (1997 ▶). For hydrogen-bond motifs, see: Bernstein *et al.* (1995 ▶). For bond-length data, see: Allen *et al.* (1987 ▶). For the stability of the temperature controller used in the data collection, see: Cosier & Glazer (1986 ▶).
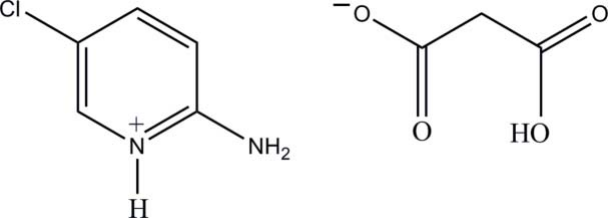


## Experimental

### 

#### Crystal data

C_5_H_6_ClN_2_^+^·C_3_H_3_O_4_^−^
*M* *_r_* = 232.62
Monoclinic, 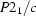

*a* = 15.6971 (19) Å
*b* = 16.866 (2) Å
*c* = 7.4662 (10) Å
β = 94.518 (3)°
*V* = 1970.5 (4) Å^3^
*Z* = 8
Mo *K*α radiation
μ = 0.38 mm^−1^
*T* = 100 K
0.22 × 0.14 × 0.07 mm

#### Data collection

Bruker APEXII DUO CCD diffractometer
Absorption correction: multi-scan (*SADABS*; Bruker, 2009 ▶) *T*_min_ = 0.921, *T*_max_ = 0.973
22474 measured reflections
5811 independent reflections
4314 reflections with *I* > 2σ(*I*)
*R*_int_ = 0.050

#### Refinement

*R*[*F* ^2^ > 2σ(*F* ^2^)] = 0.040
*wR*(*F* ^2^) = 0.097
*S* = 1.01
5811 reflections
343 parameters
H atoms treated by a mixture of independent and constrained refinement
Δρ_max_ = 0.37 e Å^−3^
Δρ_min_ = −0.29 e Å^−3^

### 

Data collection: *APEX2* (Bruker, 2009 ▶); cell refinement: *SAINT* (Bruker, 2009 ▶); data reduction: *SAINT*; program(s) used to solve structure: *SHELXTL* (Sheldrick, 2008 ▶); program(s) used to refine structure: *SHELXTL*; molecular graphics: *SHELXTL*; software used to prepare material for publication: *SHELXTL* and *PLATON* (Spek, 2009 ▶).

## Supplementary Material

Crystal structure: contains datablocks global, I. DOI: 10.1107/S1600536810019677/hb5466sup1.cif

Structure factors: contains datablocks I. DOI: 10.1107/S1600536810019677/hb5466Isup2.hkl

Additional supplementary materials:  crystallographic information; 3D view; checkCIF report

## Figures and Tables

**Table 1:** Hydrogen-bond geometry (Å, °)

*D*—H⋯*A*	*D*—H	H⋯*A*	*D*⋯*A*	*D*—H⋯*A*
N1*A*—H1*NA*⋯O1*A*	0.93 (2)	1.68 (2)	2.5982 (17)	171 (2)
N2*A*—H2*NA*⋯O2*A*	0.95 (2)	2.01 (2)	2.9518 (19)	169.1 (18)
N2*A*—H3*NA*⋯O3*B*^i^	0.87 (2)	2.07 (2)	2.9333 (18)	178 (2)
N1*B*—H1*NB*⋯O1*B*	0.92 (2)	1.69 (2)	2.5980 (17)	169 (2)
N2*B*—H2*NB*⋯O2*B*	0.88 (2)	2.08 (2)	2.9538 (19)	175 (2)
N2*B*—H3*NB*⋯O3*A*^ii^	0.93 (2)	2.04 (2)	2.9598 (19)	175 (2)
O4*A*—H1*OA*⋯O2*A*	0.94 (2)	1.58 (2)	2.4835 (16)	158 (2)
O4*B*—H1*OB*⋯O2*B*	0.93 (3)	1.57 (3)	2.4752 (16)	162 (3)
C1*A*—H1*A*⋯O3*B*^iii^	0.960 (18)	2.458 (18)	3.374 (2)	159.6 (14)
C1*B*—H1*B*⋯O3*A*^iii^	0.98 (2)	2.46 (2)	3.417 (2)	166.1 (18)
C7*A*—H7*AB*⋯O1*B*^iii^	0.97 (2)	2.31 (2)	3.2509 (19)	162.6 (18)
C7*B*—H7*BB*⋯O4*A*^iv^	0.96 (2)	2.55 (2)	3.440 (2)	155.7 (16)
C4*A*—H4*A*⋯O4*B*^i^	0.95 (2)	2.32 (2)	3.264 (2)	171.6 (18)
C4*B*—H4*B*⋯O4*A*^ii^	0.94 (2)	2.30 (2)	3.237 (2)	177.4 (17)

Symmetry codes: (i) 
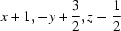
; (ii) 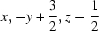; (iii) 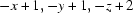; (iv) 
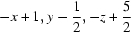
.

**Fig. 1.**
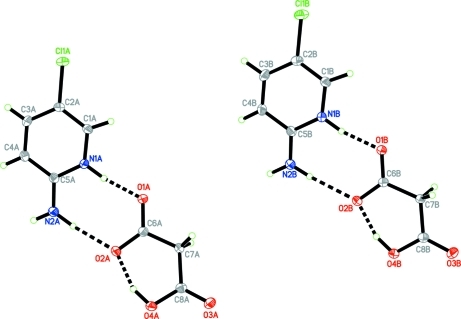
The asymmetric unit of (I). Displacement ellipsoids are drawn at the 50% probability level.

**Fig. 2.**
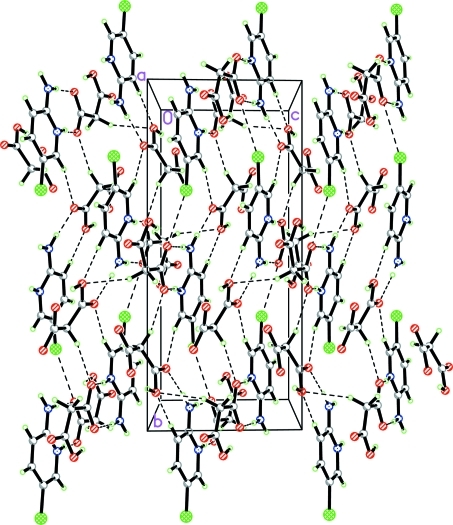
The crystal packing of (I), showing the hydrogen-bonded (dashed lines) network.

## supplementary crystallographic information

### Comment

Pyridine and its derivatives continue to attract great interest due to the wide variety of interesting biological activities observed for these compounds, such as anticancer, analgesic, antimicrobial, and antidepressant activities (Amr *et al.*, 2006; Bart *et al.*, 2001; Shinkai *et al.*, 2000; Klimesôva *et al.*, 1999). They are often involved in hydrogen-bond interactions (Jeffrey & Saenger, 1991; Jeffrey, 1997; Scheiner, 1997). The crystal structures of 2-amino-5-chloropyridine (Pourayoubi *et al.*, 2007), 2-amino-5-chloropyridinium trichloroacetate (Janczak & Perpétuo, 2009) and bis(2-amino-5-chloropyridinium)dihydrogendi phosphate (Akriche & Rzaigui, 2005) have been reported. Since our aim is to study some interesting hydrogen-bonding interactions, the crystal structure of the title salt, (I), is presented here.

The asymmetric unit of the title salt consists of two crystallographically independent 2-amino-5-chloropyridinium cations and two hydrogen malonate anions, with atom labelling suffixes of A & B (Fig. 1). Each 2-amino-5-chloropyridinium cation is planar, with a maximum deviation of 0.002 (1) Å for C5A atom (molecule A) and 0.009 (1) Å for atom N1B (molecule B).  In the cations, protonation at atoms N1A and N1B lead to slight increases in the C1A—N1A—C5A [123.22 (13)°] and C1B—N1B—C5B [122.97 (14)°] angles compared to those observed in an unprotonated structure (Pourayoubi *et al.*, 2007).  The bond lengths (Allen *et al.*, 1987) and angles are normal.

In the crystal structure, (Fig. 2), the ionic units are linked by N1A—H1NA···O1A; N2A—H2NA···O2A; N2A—H3NA···O3B; N1B—H1NB···O1B; N2B—H2NB···O2B; N2B—H3NB···O3A; C1A—H1A···O3B; C1B—H1B···O3A; C4A—H4A···O4B and C4B—H4B···O4A (Table 1) hydrogen bonds, forming one-dimensional chains along the *b*-axis. Furthermore, these chains are inter-connected by intermolecular C7A—H7AB···O1B and C7B—H7BB···O4A hydrogen bonds. There are intramolecular O4A—H1OA···O2A and O4B—H1OB···O2B hydrogen bonds in the hydrogen malonate anions, which generate *S*(6) (Bernstein *et al.*, 1995) ring motifs, resulting in folded conformation.

### Experimental

A hot methanol solution (20 ml) of 2-amino-5-chloropyridine (64 mg, Aldrich) and malonic acid acid (52 mg, Merck) were mixed and warmed over a heating magnetic stirrer hotplate for a few minutes. The resulting solution was allowed to cool slowly at room temperature and colourless needles of (I) appeared after a few days.

### Refinement

All the H atoms were located from the difference Fourier maps and allowed to refine freely [N–H = 0.87 (2)–0.95 (2) Å, O–H = 0.93 (3)–0.94 (3) Å and C–H = 0.90 (2)–0.97 (2) Å].

### Crystal data

**Table:**

C_5_H_6_ClN_2_^+^·C_3_H_3_O_4_^−^	*F*(000) = 960
*M**_r_* = 232.62	*D*_x_ = 1.568 Mg m^−^^3^
Monoclinic, *P*2_1_/*c*	Mo *K*α radiation, λ = 0.71073 Å
Hall symbol: -P 2ybc	Cell parameters from 3966 reflections
*a* = 15.6971 (19) Å	θ = 2.6–29.5°
*b* = 16.866 (2) Å	µ = 0.38 mm^−^^1^
*c* = 7.4662 (10) Å	*T* = 100 K
β = 94.518 (3)°	Needle, colourless
*V* = 1970.5 (4) Å^3^	0.22 × 0.14 × 0.07 mm
*Z* = 8	

### Data collection

**Table:**

Bruker APEXII DUO CCD diffractometer	5811 independent reflections
Radiation source: fine-focus sealed tube	4314 reflections with *I* > 2σ(*I*)
graphite	*R*_int_ = 0.050
φ and ω scans	θ_max_ = 30.1°, θ_min_ = 1.3°
Absorption correction: multi-scan (*SADABS*; Bruker, 2009)	*h* = −22→22
*T*_min_ = 0.921, *T*_max_ = 0.973	*k* = −23→23
22474 measured reflections	*l* = −10→10

### Refinement

**Table:**

Refinement on *F*^2^	Primary atom site location: structure-invariant direct methods
Least-squares matrix: full	Secondary atom site location: difference Fourier map
*R*[*F*^2^ > 2σ(*F*^2^)] = 0.040	Hydrogen site location: inferred from neighbouring sites
*wR*(*F*^2^) = 0.097	H atoms treated by a mixture of independent and constrained refinement
*S* = 1.01	*w* = 1/[σ^2^(*F*_o_^2^) + (0.0434*P*)^2^ + 0.3626*P*] where *P* = (*F*_o_^2^ + 2*F*_c_^2^)/3
5811 reflections	(Δ/σ)_max_ = 0.001
343 parameters	Δρ_max_ = 0.37 e Å^−^^3^
0 restraints	Δρ_min_ = −0.29 e Å^−^^3^

### Special details

**Table:**

Experimental. The crystal was placed in the cold stream of an Oxford Cryosystems Cobra open-flow nitrogen cryostat (Cosier & Glazer, 1986) operating at 100.0 (1) K.
Geometry. All s.u.'s (except the s.u. in the dihedral angle between two l.s. planes) are estimated using the full covariance matrix. The cell s.u.'s are taken into account individually in the estimation of s.u.'s in distances, angles and torsion angles; correlations between s.u.'s in cell parameters are only used when they are defined by crystal symmetry. An approximate (isotropic) treatment of cell s.u.'s is used for estimating s.u.'s involving l.s. planes.
Refinement. Refinement of F^2^ against ALL reflections. The weighted R-factor wR and goodness of fit S are based on F^2^, conventional R-factors R are based on F, with F set to zero for negative F^2^. The threshold expression of F^2^ > 2σ(F^2^) is used only for calculating R-factors(gt) etc. and is not relevant to the choice of reflections for refinement. R-factors based on F^2^ are statistically about twice as large as those based on F, and R- factors based on ALL data will be even larger.

### Fractional atomic coordinates and isotropic or equivalent isotropic displacement parameters (Å^2^)

**Table:**

	*x*	*y*	*z*	*U*_iso_*/*U*_eq_	
Cl1A	1.10339 (2)	0.51132 (2)	0.62925 (6)	0.02059 (10)	
N1A	0.96753 (8)	0.70248 (8)	0.71311 (18)	0.0154 (3)	
N2A	0.98853 (9)	0.83754 (8)	0.6948 (2)	0.0201 (3)	
C1A	0.99204 (10)	0.62574 (9)	0.7005 (2)	0.0158 (3)	
C2A	1.07092 (10)	0.60860 (9)	0.6478 (2)	0.0161 (3)	
C3A	1.12584 (10)	0.67048 (10)	0.6084 (2)	0.0185 (3)	
C4A	1.10044 (10)	0.74747 (9)	0.6221 (2)	0.0183 (3)	
C5A	1.01808 (10)	0.76410 (9)	0.6772 (2)	0.0153 (3)	
O1A	0.80991 (7)	0.72010 (7)	0.78892 (17)	0.0217 (3)	
O2A	0.82767 (7)	0.84610 (6)	0.87375 (16)	0.0188 (2)	
O3A	0.59603 (7)	0.85002 (7)	1.08754 (16)	0.0210 (3)	
O4A	0.71363 (7)	0.91411 (7)	1.02782 (17)	0.0202 (2)	
C6A	0.78450 (10)	0.78253 (9)	0.8570 (2)	0.0152 (3)	
C7A	0.69498 (10)	0.78000 (9)	0.9200 (2)	0.0150 (3)	
C8A	0.66443 (10)	0.85090 (9)	1.0200 (2)	0.0156 (3)	
Cl1B	0.63656 (3)	0.19876 (2)	0.73072 (6)	0.02333 (10)	
N1B	0.49352 (8)	0.38598 (8)	0.80976 (18)	0.0157 (3)	
N2B	0.50075 (9)	0.51989 (8)	0.7476 (2)	0.0208 (3)	
C1B	0.52319 (10)	0.31042 (9)	0.8071 (2)	0.0164 (3)	
C2B	0.59873 (10)	0.29500 (10)	0.7381 (2)	0.0175 (3)	
C3B	0.64554 (10)	0.35768 (10)	0.6699 (2)	0.0200 (3)	
C4B	0.61446 (10)	0.43307 (10)	0.6713 (2)	0.0192 (3)	
C5B	0.53518 (10)	0.44787 (9)	0.7429 (2)	0.0162 (3)	
O1B	0.34096 (7)	0.39509 (6)	0.92022 (17)	0.0215 (3)	
O2B	0.33226 (7)	0.52644 (6)	0.89745 (17)	0.0209 (3)	
O3B	0.10788 (7)	0.53283 (7)	1.13690 (18)	0.0270 (3)	
O4B	0.21003 (7)	0.59797 (7)	1.01198 (17)	0.0213 (3)	
C6B	0.30412 (10)	0.45958 (9)	0.9424 (2)	0.0157 (3)	
C7B	0.22061 (10)	0.45532 (9)	1.0316 (2)	0.0163 (3)	
C8B	0.17473 (10)	0.53207 (9)	1.0644 (2)	0.0172 (3)	
H1A	0.9511 (11)	0.5865 (11)	0.730 (2)	0.016 (4)*	
H1B	0.4871 (12)	0.2697 (12)	0.856 (3)	0.026 (5)*	
H3A	1.1788 (13)	0.6612 (11)	0.572 (3)	0.025 (5)*	
H3B	0.7003 (13)	0.3485 (11)	0.623 (3)	0.026 (5)*	
H4A	1.1372 (13)	0.7899 (12)	0.597 (3)	0.028 (5)*	
H4B	0.6435 (13)	0.4767 (12)	0.626 (3)	0.026 (5)*	
H7AA	0.6570 (12)	0.7744 (11)	0.815 (3)	0.023 (5)*	
H7AB	0.6906 (13)	0.7322 (12)	0.991 (3)	0.029 (5)*	
H7BA	0.1847 (14)	0.4223 (13)	0.967 (3)	0.035 (6)*	
H7BB	0.2294 (13)	0.4291 (12)	1.145 (3)	0.031 (6)*	
H1NA	0.9120 (14)	0.7144 (13)	0.738 (3)	0.035 (6)*	
H2NA	0.9348 (14)	0.8461 (12)	0.743 (3)	0.033 (6)*	
H3NA	1.0235 (14)	0.8762 (14)	0.680 (3)	0.041 (6)*	
H1NB	0.4410 (14)	0.3956 (12)	0.851 (3)	0.034 (6)*	
H2NB	0.4509 (13)	0.5249 (12)	0.793 (3)	0.028 (5)*	
H3NB	0.5290 (14)	0.5628 (14)	0.702 (3)	0.038 (6)*	
H1OA	0.7643 (16)	0.8995 (15)	0.976 (3)	0.051 (7)*	
H1OB	0.2602 (17)	0.5811 (16)	0.965 (4)	0.058 (8)*	

### Atomic displacement parameters (Å^2^)

**Table:**

	*U*^11^	*U*^22^	*U*^33^	*U*^12^	*U*^13^	*U*^23^
Cl1A	0.02137 (19)	0.01383 (18)	0.0268 (2)	0.00466 (14)	0.00314 (16)	−0.00142 (16)
N1A	0.0130 (6)	0.0137 (6)	0.0202 (7)	−0.0002 (5)	0.0045 (5)	−0.0004 (5)
N2A	0.0188 (7)	0.0131 (7)	0.0292 (8)	−0.0008 (5)	0.0074 (6)	0.0005 (6)
C1A	0.0172 (7)	0.0120 (7)	0.0182 (7)	−0.0011 (6)	0.0025 (6)	−0.0002 (6)
C2A	0.0172 (7)	0.0128 (7)	0.0182 (7)	0.0019 (5)	0.0016 (6)	−0.0007 (6)
C3A	0.0134 (7)	0.0198 (8)	0.0228 (8)	0.0008 (6)	0.0054 (6)	−0.0010 (7)
C4A	0.0161 (7)	0.0156 (7)	0.0238 (8)	−0.0030 (6)	0.0054 (6)	0.0015 (6)
C5A	0.0165 (7)	0.0147 (7)	0.0149 (7)	−0.0015 (5)	0.0018 (6)	0.0004 (6)
O1A	0.0173 (5)	0.0146 (5)	0.0346 (7)	−0.0007 (4)	0.0101 (5)	−0.0051 (5)
O2A	0.0185 (5)	0.0132 (5)	0.0255 (6)	−0.0037 (4)	0.0074 (5)	−0.0022 (5)
O3A	0.0193 (5)	0.0195 (6)	0.0250 (6)	0.0019 (4)	0.0080 (5)	−0.0011 (5)
O4A	0.0225 (6)	0.0123 (5)	0.0272 (6)	−0.0009 (4)	0.0096 (5)	−0.0026 (5)
C6A	0.0155 (7)	0.0137 (7)	0.0165 (7)	0.0004 (5)	0.0023 (6)	0.0013 (6)
C7A	0.0150 (7)	0.0122 (7)	0.0178 (7)	−0.0013 (5)	0.0023 (6)	−0.0001 (6)
C8A	0.0187 (7)	0.0128 (7)	0.0154 (7)	0.0016 (5)	0.0026 (6)	0.0025 (6)
Cl1B	0.0262 (2)	0.0190 (2)	0.0250 (2)	0.00833 (15)	0.00331 (16)	0.00035 (17)
N1B	0.0148 (6)	0.0144 (6)	0.0184 (6)	−0.0006 (5)	0.0038 (5)	0.0001 (5)
N2B	0.0198 (7)	0.0146 (7)	0.0288 (8)	−0.0001 (5)	0.0081 (6)	0.0015 (6)
C1B	0.0177 (7)	0.0140 (7)	0.0174 (7)	−0.0003 (6)	0.0011 (6)	0.0013 (6)
C2B	0.0191 (7)	0.0166 (7)	0.0164 (7)	0.0037 (6)	−0.0006 (6)	−0.0007 (6)
C3B	0.0161 (7)	0.0249 (8)	0.0195 (8)	0.0010 (6)	0.0053 (6)	−0.0024 (7)
C4B	0.0176 (7)	0.0194 (8)	0.0212 (8)	−0.0032 (6)	0.0061 (6)	−0.0009 (7)
C5B	0.0176 (7)	0.0148 (7)	0.0162 (7)	−0.0025 (6)	0.0015 (6)	−0.0010 (6)
O1B	0.0192 (5)	0.0124 (5)	0.0342 (7)	0.0012 (4)	0.0110 (5)	0.0018 (5)
O2B	0.0208 (6)	0.0119 (5)	0.0312 (7)	−0.0005 (4)	0.0094 (5)	0.0013 (5)
O3B	0.0246 (6)	0.0194 (6)	0.0392 (8)	0.0045 (5)	0.0157 (6)	0.0017 (6)
O4B	0.0199 (6)	0.0128 (5)	0.0321 (7)	0.0012 (4)	0.0082 (5)	−0.0002 (5)
C6B	0.0162 (7)	0.0137 (7)	0.0175 (7)	0.0013 (5)	0.0024 (6)	−0.0002 (6)
C7B	0.0170 (7)	0.0113 (7)	0.0212 (8)	0.0015 (5)	0.0054 (6)	0.0016 (6)
C8B	0.0178 (7)	0.0147 (7)	0.0193 (8)	0.0020 (6)	0.0016 (6)	0.0005 (6)

### Geometric parameters (Å, °)

**Table:**

Cl1A—C2A	1.7268 (16)	Cl1B—C2B	1.7308 (16)
N1A—C5A	1.3472 (19)	N1B—C5B	1.3484 (19)
N1A—C1A	1.3557 (19)	N1B—C1B	1.358 (2)
N1A—H1NA	0.93 (2)	N1B—H1NB	0.92 (2)
N2A—C5A	1.333 (2)	N2B—C5B	1.331 (2)
N2A—H2NA	0.95 (2)	N2B—H2NB	0.88 (2)
N2A—H3NA	0.87 (2)	N2B—H3NB	0.93 (2)
C1A—C2A	1.360 (2)	C1B—C2B	1.355 (2)
C1A—H1A	0.959 (18)	C1B—H1B	0.98 (2)
C2A—C3A	1.400 (2)	C2B—C3B	1.406 (2)
C3A—C4A	1.365 (2)	C3B—C4B	1.362 (2)
C3A—H3A	0.91 (2)	C3B—H3B	0.96 (2)
C4A—C5A	1.415 (2)	C4B—C5B	1.415 (2)
C4A—H4A	0.95 (2)	C4B—H4B	0.94 (2)
O1A—C6A	1.2481 (18)	O1B—C6B	1.2491 (18)
O2A—C6A	1.2692 (18)	O2B—C6B	1.2660 (19)
O3A—C8A	1.2217 (19)	O3B—C8B	1.2181 (19)
O4A—C8A	1.3150 (18)	O4B—C8B	1.3153 (19)
O4A—H1OA	0.94 (3)	O4B—H1OB	0.93 (3)
C6A—C7A	1.517 (2)	C6B—C7B	1.518 (2)
C7A—C8A	1.508 (2)	C7B—C8B	1.511 (2)
C7A—H7AA	0.95 (2)	C7B—H7BA	0.90 (2)
C7A—H7AB	0.97 (2)	C7B—H7BB	0.95 (2)
			
C5A—N1A—C1A	123.22 (13)	C5B—N1B—C1B	122.97 (14)
C5A—N1A—H1NA	116.7 (13)	C5B—N1B—H1NB	117.6 (13)
C1A—N1A—H1NA	119.8 (13)	C1B—N1B—H1NB	119.3 (13)
C5A—N2A—H2NA	120.2 (13)	C5B—N2B—H2NB	118.2 (13)
C5A—N2A—H3NA	117.3 (15)	C5B—N2B—H3NB	119.7 (14)
H2NA—N2A—H3NA	121.5 (19)	H2NB—N2B—H3NB	122.1 (19)
N1A—C1A—C2A	119.54 (14)	C2B—C1B—N1B	119.84 (15)
N1A—C1A—H1A	116.4 (11)	C2B—C1B—H1B	123.8 (12)
C2A—C1A—H1A	124.1 (11)	N1B—C1B—H1B	116.4 (12)
C1A—C2A—C3A	119.49 (14)	C1B—C2B—C3B	119.44 (15)
C1A—C2A—Cl1A	120.43 (12)	C1B—C2B—Cl1B	120.34 (13)
C3A—C2A—Cl1A	120.07 (12)	C3B—C2B—Cl1B	120.22 (12)
C4A—C3A—C2A	120.35 (14)	C4B—C3B—C2B	120.10 (15)
C4A—C3A—H3A	117.8 (12)	C4B—C3B—H3B	118.8 (12)
C2A—C3A—H3A	121.9 (12)	C2B—C3B—H3B	121.1 (12)
C3A—C4A—C5A	119.31 (14)	C3B—C4B—C5B	119.59 (15)
C3A—C4A—H4A	121.2 (12)	C3B—C4B—H4B	122.9 (12)
C5A—C4A—H4A	119.5 (12)	C5B—C4B—H4B	117.5 (12)
N2A—C5A—N1A	118.84 (14)	N2B—C5B—N1B	119.12 (14)
N2A—C5A—C4A	123.08 (14)	N2B—C5B—C4B	122.84 (15)
N1A—C5A—C4A	118.08 (14)	N1B—C5B—C4B	118.04 (14)
C8A—O4A—H1OA	106.3 (15)	C8B—O4B—H1OB	104.0 (16)
O1A—C6A—O2A	124.57 (14)	O1B—C6B—O2B	124.45 (14)
O1A—C6A—C7A	115.85 (13)	O1B—C6B—C7B	116.22 (13)
O2A—C6A—C7A	119.57 (13)	O2B—C6B—C7B	119.33 (13)
C8A—C7A—C6A	118.02 (13)	C8B—C7B—C6B	118.02 (13)
C8A—C7A—H7AA	106.5 (12)	C8B—C7B—H7BA	109.3 (13)
C6A—C7A—H7AA	106.5 (12)	C6B—C7B—H7BA	108.6 (13)
C8A—C7A—H7AB	110.6 (12)	C8B—C7B—H7BB	106.9 (12)
C6A—C7A—H7AB	107.4 (12)	C6B—C7B—H7BB	109.9 (12)
H7AA—C7A—H7AB	107.4 (16)	H7BA—C7B—H7BB	103.1 (18)
O3A—C8A—O4A	121.58 (14)	O3B—C8B—O4B	121.41 (14)
O3A—C8A—C7A	121.26 (14)	O3B—C8B—C7B	121.29 (14)
O4A—C8A—C7A	117.15 (13)	O4B—C8B—C7B	117.29 (13)
			
C5A—N1A—C1A—C2A	−0.6 (2)	C5B—N1B—C1B—C2B	1.4 (2)
N1A—C1A—C2A—C3A	0.4 (2)	N1B—C1B—C2B—C3B	0.0 (2)
N1A—C1A—C2A—Cl1A	−179.58 (12)	N1B—C1B—C2B—Cl1B	−179.02 (12)
C1A—C2A—C3A—C4A	−0.2 (3)	C1B—C2B—C3B—C4B	−0.8 (3)
Cl1A—C2A—C3A—C4A	179.75 (14)	Cl1B—C2B—C3B—C4B	178.16 (13)
C2A—C3A—C4A—C5A	0.2 (3)	C2B—C3B—C4B—C5B	0.4 (3)
C1A—N1A—C5A—N2A	−179.49 (15)	C1B—N1B—C5B—N2B	178.66 (15)
C1A—N1A—C5A—C4A	0.6 (2)	C1B—N1B—C5B—C4B	−1.7 (2)
C3A—C4A—C5A—N2A	179.68 (17)	C3B—C4B—C5B—N2B	−179.60 (16)
C3A—C4A—C5A—N1A	−0.4 (2)	C3B—C4B—C5B—N1B	0.8 (2)
O1A—C6A—C7A—C8A	173.93 (14)	O1B—C6B—C7B—C8B	178.82 (15)
O2A—C6A—C7A—C8A	−6.9 (2)	O2B—C6B—C7B—C8B	−0.5 (2)
C6A—C7A—C8A—O3A	−173.71 (15)	C6B—C7B—C8B—O3B	−179.34 (16)
C6A—C7A—C8A—O4A	7.8 (2)	C6B—C7B—C8B—O4B	0.5 (2)

### Hydrogen-bond geometry (Å, °)

**Table:**

*D*—H···*A*	*D*—H	H···*A*	*D*···*A*	*D*—H···*A*
N1A—H1NA···O1A	0.93 (2)	1.68 (2)	2.5982 (17)	171 (2)
N2A—H2NA···O2A	0.95 (2)	2.01 (2)	2.9518 (19)	169.1 (18)
N2A—H3NA···O3B^i^	0.87 (2)	2.07 (2)	2.9333 (18)	178 (2)
N1B—H1NB···O1B	0.92 (2)	1.69 (2)	2.5980 (17)	169 (2)
N2B—H2NB···O2B	0.88 (2)	2.08 (2)	2.9538 (19)	175 (2)
N2B—H3NB···O3A^ii^	0.93 (2)	2.04 (2)	2.9598 (19)	175 (2)
O4A—H1OA···O2A	0.94 (2)	1.58 (2)	2.4835 (16)	158 (2)
O4B—H1OB···O2B	0.93 (3)	1.57 (3)	2.4752 (16)	162 (3)
C1A—H1A···O3B^iii^	0.960 (18)	2.458 (18)	3.374 (2)	159.6 (14)
C1B—H1B···O3A^iii^	0.98 (2)	2.46 (2)	3.417 (2)	166.1 (18)
C7A—H7AB···O1B^iii^	0.97 (2)	2.31 (2)	3.2509 (19)	162.6 (18)
C7B—H7BB···O4A^iv^	0.96 (2)	2.55 (2)	3.440 (2)	155.7 (16)
C4A—H4A···O4B^i^	0.95 (2)	2.32 (2)	3.264 (2)	171.6 (18)
C4B—H4B···O4A^ii^	0.94 (2)	2.30 (2)	3.237 (2)	177.4 (17)

Symmetry codes: (i) *x*+1, −*y*+3/2, *z*−1/2; (ii) *x*, −*y*+3/2, *z*−1/2; (iii) −*x*+1, −*y*+1, −*z*+2; (iv) −*x*+1, *y*−1/2, −*z*+5/2.

## References

[bb1] Akriche, S. & Rzaigui, M. (2005). *Acta Cryst.* E**61**, o2607–o2609.

[bb2] Allen, F. H., Kennard, O., Watson, D. G., Brammer, L., Orpen, A. G. & Taylor, R. (1987). *J. Chem. Soc. Perkin Trans. 2*, pp. S1–19.

[bb3] Amr, A. G., Mohamed, A. M., Mohamed, S. F., Abdel-Hafez, N. A. & Hammam, A. G. (2006). *Bioorg. Med. Chem.* **14**, 5481–5488. 10.1016/j.bmc.2006.04.045 16713269

[bb4] Bart, A., Jansen, J., Zwan, J. V., Dulk, H., Brouwer, J. & Reedijk, J. (2001). *J. Med. Chem.* **44**, 245–249. 10.1021/jm0010163 11170634

[bb5] Bernstein, J., Davis, R. E., Shimoni, L. & Chang, N.-L. (1995). *Angew. Chem. Int. Ed. Engl.* **34**, 1555–1573.

[bb6] Bruker (2009). *APEX2*, *SAINT* and *SADABS* Bruker AXS Inc., Madison, Wisconsin, USA.

[bb7] Cosier, J. & Glazer, A. M. (1986). *J. Appl. Cryst.* **19**, 105–107.

[bb8] Janczak, J. & Perpétuo, G. J. (2009). *Acta Cryst.* C**65**, o339–o341. 10.1107/S0108270109021490 19578268

[bb9] Jeffrey, G. A. (1997). *An Introduction to Hydrogen Bonding.* Oxford University Press.

[bb10] Jeffrey, G. A. & Saenger, W. (1991). *Hydrogen Bonding in Biological Structures.* Berlin: Springer.

[bb11] Klimesôva, V., Svoboda, M., Waisser, K., Pour, M. & Kaustova, J. (1999). *Il Farmaco*, **54**, 666–672. 10.1016/s0014-827x(99)00078-6 10575735

[bb12] Pourayoubi, M., Ghadimi, S. & Ebrahimi Valmoozi, A. A. (2007). *Acta Cryst.* E**63**, o4631. 10.1107/S1600536810002692 PMC2979840 21579865

[bb13] Scheiner, S. (1997). *Hydrogen Bonding. A Theoretical Perspective.* Oxford University Press.

[bb14] Sheldrick, G. M. (2008). *Acta Cryst.* A**64**, 112–122. 10.1107/S0108767307043930 18156677

[bb15] Shinkai, H., Ito, T., Iida, T., Kitao, Y., Yamada, H. & Uchida, I. (2000). *J. Med. Chem.* **43**, 4667–4677. 10.1021/jm0002073 11101358

[bb16] Spek, A. L. (2009). *Acta Cryst.* D**65**, 148–155. 10.1107/S090744490804362X PMC2631630 19171970

